# Finite element analysis of mechanics of neovessels with intraplaque hemorrhage in carotid atherosclerosis

**DOI:** 10.1186/1475-925X-14-S1-S3

**Published:** 2015-01-09

**Authors:** Jinqiu Lu, Wanying Duan, Aike Qiao

**Affiliations:** 1 College of Life Science and Bioengineering, Beijing University of Technology, Beijing, 100124, China; 2 Neural Department of Internal Medicine, Beijing Tian Tan Hospital, Capital Medical University, Beijing, 100050, China

## Abstract

**Background:**

Intraplaque hemorrhage is a widely known factor facilitating plaque instability. Neovascularization of plaque can be regarded as a compensatory response to the blood supply in the deep intimal and medial areas of the artery. Due to the physiological function, the deformation of carotid atherosclerotic plaque would happen under the action of blood pressure and blood flow. Neovessels are subject to mechanical loading and likely undergo deformation. The rupture of neovessels may deteriorate the instability of plaque. This study focuses on the local mechanical environments around neovessels and investigates the relationship between the biomechanics and the morphological specificity of neovessels.

**Methods:**

Stress and stretch were used to evaluate the rupture risk of the neovessels in plaque. Computational structural analysis was performed based on two human carotid plaque slice samples. Two-dimensional models containing neovessels and other components were built according to the plaque slice samples. Each component was assumed to be non-linear isotropic, piecewise homogeneous and incompressible. Different mechanical boundary conditions, i.e. static pressures, were imposed in the carotid lumen and neovessels lumen respectively. Finite element method was used to simulate the mechanical conditions in the atherosclerotic plaque.

**Results:**

Those neovessels closer to the carotid lumen undergo larger stress and stretch. With the same distance to the carotid lumen, the longer the perimeter of neovessels is, the larger stress and the deformation of the neovessels will be. Under the same conditions, the neovessels with larger curvature suffer greater stress and stretch. Neovessels surrounded by red blood cells undergo a much larger stretch.

**Conclusions:**

Local mechanical conditions may result in the hemorrhage of neovessels and accelerate the rupture of plaque. The mechanical environments of the neovessel are related to its shape, curvature, distance to the carotid lumen and the material properties of plaque.

## Background

According to the medical statistics, stroke (either ischemic or hemorrhagic) is the third leading cause of death and the primary cause of disability in the world [1,2]. In western countries, about 80% to 85% of strokes among adults are ischemic [3]. Most of the ischemic strokes are caused by the blockage in an artery that supplies blood to the brain, and hence result in a deficiency in blood flow (ischemia).

Atherosclerotic plaque rupture is the main cause of stroke and may occur without any warning [4-7]. In the process of development and operation, atherosclerotic plaques may suddenly rupture, causing plaque debris flow and intraluminal thrombosis. Researches have shown that plaque instability is caused by cerebral infarction on the nervous system, such as a risk factor for severe damage [8]. So it is very important to judge the stability of atherosclerotic plaque for the prevention and treatment of vital stroke. Nevertheless, clinical assessment of stroke risk is still mainly based on the degree of luminal stenosis severity as measured [9]. However, more and more evidences suggest that degree of luminal stenosis alone is insufficient for identifying the critical condition [10].

Studies have demonstrated the correlation between large lipid rich necrotic core with a thin or ruptured fibrous and atherosclerotic plaque rupture [11]. Some other factors, such as plaque inflammation, fissured plaque, sex differences and intraplaque hemorrhage, are also considered [12-15]. Studies found that in the event of a plaque in patients with rupture hemorrhage caused by plaque, the detection rate of neovessels is very high [16,17]. Besides these factors, the mechanism of reducing plaque stability is unspecified for the neovessels in the plaque under physiological conditions. Pathological neovessel can be identified in early atherosclerosis. There is growing number of evidences suggesting that intraplaque neovessels are closely associated with intraplaque hemorrhage (IHP). But how do intraplaque neovessels promote IPH needs further investigation.

Finite element method is widely used in the biomechanical field. It can be used to predict plaque vulnerability based on peak plaque stress using human samples [18]. By using finite element method, computational models combing mechanical factors and morphologic information can be employed to implement plaque mechanical analysis, and identify additional critical mechanical factors so as to improve the current assessment criteria of plaque vulnerability based on histology and image [19-23]. Teng et al performed finite element analysis of mechanics in plaque with neovessels and showed that there are large degrees of deformation and high variation in the mechanical loading around intraplaque neovessels during the cardiac cycle [24]. Finite element analysis method can be used to quantify the critical mechanical conditions around neovessels and characterize the association between these conditions and plaque's pathological features, such as the distribution of red blood cells (RBCs) as a marker of IHP. Experimental studies have repeatedly confirmed that ischemia hypoxia is the basic cause of intraplaque angiogenesis [25,26], while there is no specific law to follow about the size and shape of the neovessels.

The objective of this study is to further investigate the relationship between the critical mechanical conditions (stress and stretch) around neovessels with the morphological specificity (perimeter and curvature) and the distance to the main vessel lumen. The purpose of this paper is to evaluate the stability of plaque and provide a new way for the clinical assessment of stroke risk.

## Material and methods

**Figure 1:**
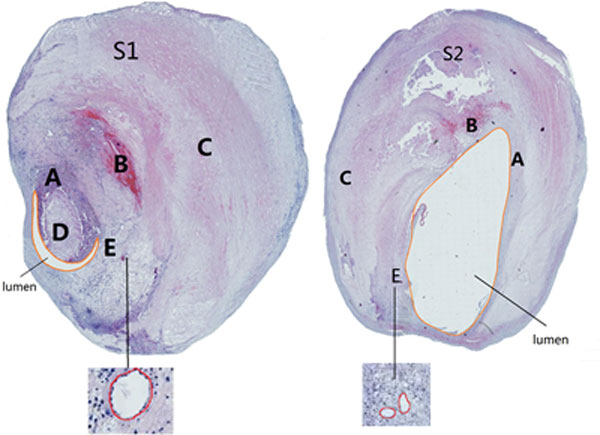
**Two samples of microscopic slices of plaque stained using H&E**. (Left: S1; Right: S2). A: Fibrous cap; B: Fresh intraplaque hemorrhage; C: Vessel; D: Lipid core; E: Neovessels.

The present study was performed using computational structural analysis based on two carotid plaque samples which were collected with endarterectomy for histopathological examination from Department of Neurology, Beijing Tian Tan Hospital, with patient consent obtained. One of them contains lipid core while another not. In the process of staining, the lipid core occurred shedding in S2 (sample 2). The patient's blood pressures were 159mmHg and 140mmHg at systole respectively. The samples were formalin-saline fixed, decalcified, embedded in paraffin and stained using hematoxylin and eosin (H&E), and then stained using Platelet endothelial cell adhesion molecule-1(PECAM-1/CD31) or Actin alpha, smooth muscle aorta (α-SMA). Both S1(sample1) and S2(sample 2) are decalcification. Figure 1 shows the histological slice stained using H&E. There are a great number of neovessels (Figure 1E) in the samples (which can be seen in the slice stained by α-SMA). All contours were manually traced by using Motic DSAssistant Lite (Motic, Inc., Amoy). Because it is a manual operation, we do not deny that there are some tiny random errors. Those contours are lumen borders and plaque components.

AutoCAD (Autodesk, Inc., USA) was applied to establish the two-dimensional models of the H&E slices containing neovessels, fibrous cap, lipid core, fresh IPH and other components called vascular area. All components were modeled as nonlinear hyper-elastic, piecewise homogeneous and incompressible materials. Mooney-Rivlin model was used to describe the material properties of the plaque [23,27]. Materials properties are governed by the strain energy density function (Eq. 1).

(1) $$\text{W}={\text{C}}_{1}\left({\text{I}}_{1}-3\right)+{\text{D}}_{1}\text{exp}\left[{\text{D}}_{2}\left({\text{I}}_{1}-3\right)-1\right],$$

where I_1 _is the first strain invariant and C_1_, D_1 _and D_2 _are coefficients of materials respectively. The details of these coefficients are shown in Table 1 according to the studies of Teng et al. [24,28]

**Table 1:** Coefficients of strain energy density function.

Components	C_1 _(kPa)	D_1 _(kPa)	D_2_
Vessel material	36.8	14.4	2
Fibrous cap	73.6	28.8	2.5
Lipid core	2	2	1.5
Fresh IPH	1	1	0.25

**Figure 2:**
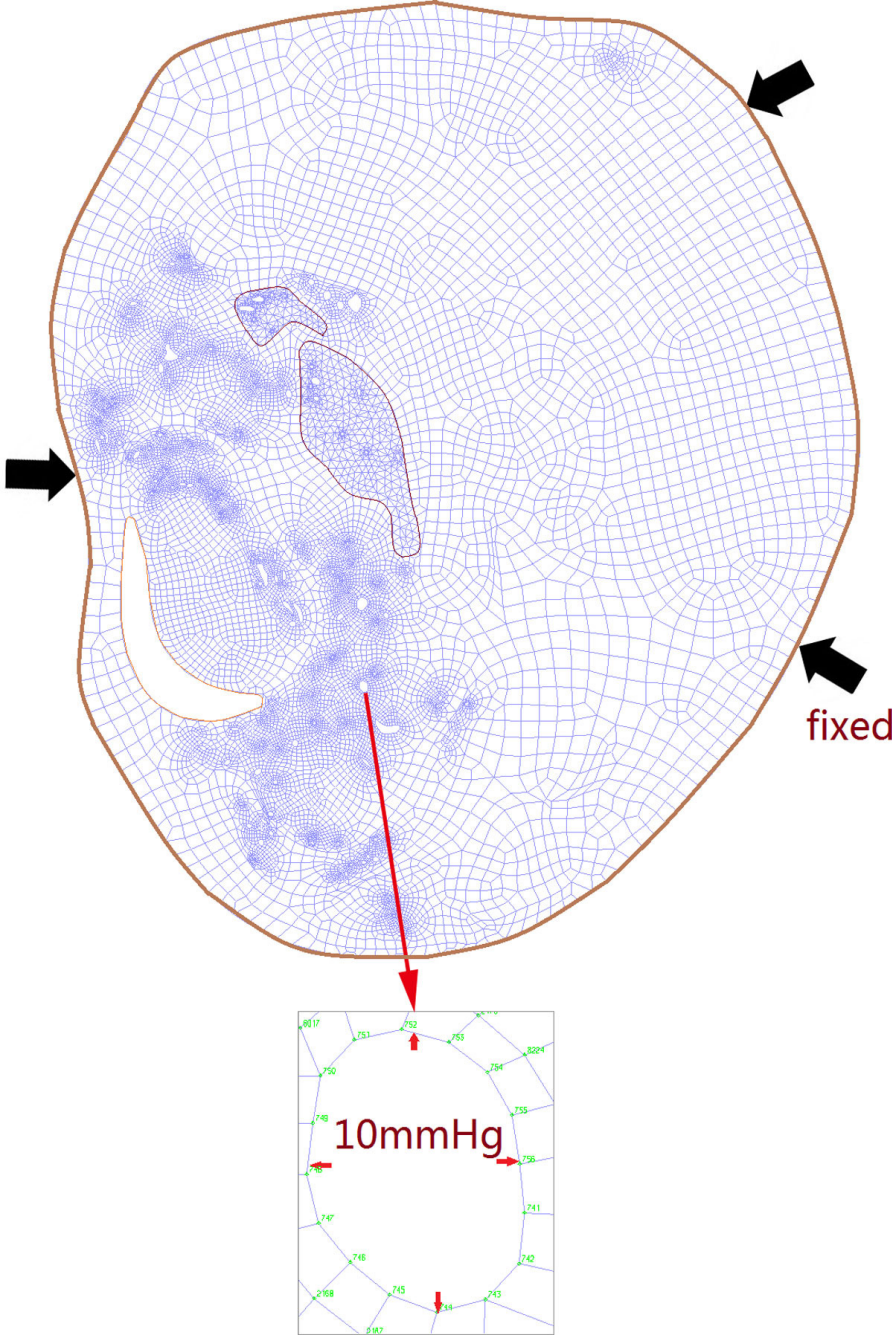
**The 2D models for finite element analysis**.

The blood pressures at systole of the patients (159 mmHg and 140 mmHg) were provided by the Department of Neurology, Beijing Tian Tan Hospital. Finite element analyses of static structural mechanics of atherosclerotic plaque under physiological loading were performed using package ADINA8.8.1 (ADINA R&D, Inc., USA). Two cases with different blood pressures of patients at systole were assigned to the main vessel lumen of carotid artery, and the pressure in each neovessel was assumed to be 10 mmHg. "This value was chosen because it approximately reflects blood pressure in the venous environment. The experimental conclusions did not change when the value was lowered to 5 mmHg" [24]. The plaque is located inside the vessel and the carotid artery is surrounded by a lot of soft tissue, so we restricted the external displacement of plaque. Boundary conditions are illustrated in Figure 2.

In order to get more accurate results, the neovessels were divided into two groups based on its location (We checked the stress and stretch based on the grid numbers of element, as shown in Figure 2), i.e. one group in IPH area with-RBC surrounded and another group in area without-RBC surrounded. Each neovessel outline was divided into more than 10 elements, There are more than 10000 elements in both two samples. Using the MATLAB (Mathworks, Inc., USA) to calculate the shortest distance between each point and the lumen, and the minimum value was defined as the distance between neovessel and lumen. The coordinates of three adjacent points were used to calculate the curvature of the neovessels, and the maximum value of curvature was taken for further results exhibition and discussion. The association between this geometric distribution and critical mechanical condition was further analyzed.

## Results

There are 167 neovessels identified in S1, 42 of them (25%) locate in the fresh IPH area and 125 neovessels are identified in S2, 20 of them locate in the fresh IPH area. The emphases are focused on the effects of the distance to the lumen, the size and curvature of neovessels and vessel material properties, respectively.

### Effect of distance to lumen

**Figure 3:**
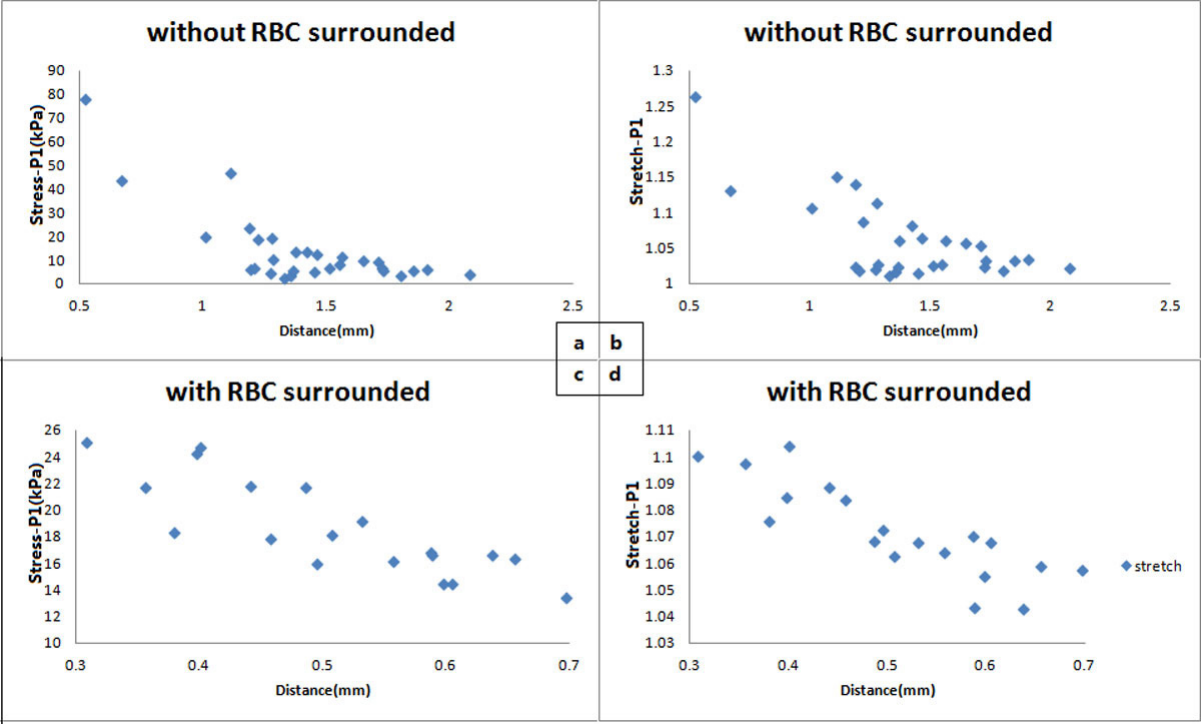
**The relationship between critical mechanical conditions around the neovessels and their distance from the main arterial lumen**. (a) local maximum principal stress (Stress-P1) at systole when neovessels without red blood cells surrounded; (b) local maximum principal stretch (Stretch-P1) at systole when neovessels without red blood cells surrounded; (c) local maximum principal stress (Stress-P1) at systole when neovessels with red blood cells surrounded; (d) local maximum principal stretch (Stretch-P1) at systole when neovessels with red blood cells surrounded.

According to the distance to the lumen of the neovessels, the mechanical situations of neovessels are particularly considered. The analysis of the mechanical conditions of neovessels in plaque will be demonstrated. Two groups of neovessels with similar curvature and similar perimeter are randomly selected. The relationships between the local maximum principal stress Stress-P_1 _and the local maximum principal stretch Stretch-P_1 _with the various distances to lumen of each neovessel are shown in Figure 3.

With the increase of the distance between neovessel and lumen, the Stress-P_1 _and Stretch-P_1 _for each neovessel are decreased. The mechanical condition for intraplaque neovessels is affected by the pressure of carotid artery. Therefore, it can be deduced that for the neovessel located in the region of the same material, the closer to the lumen, the greater risk of plaque rupture. The same result can be observed from the other groups with similar curvature and perimeter, and from both samples whether located in IPH area or not.

### Effect of size of vessels

**Figure 4:**
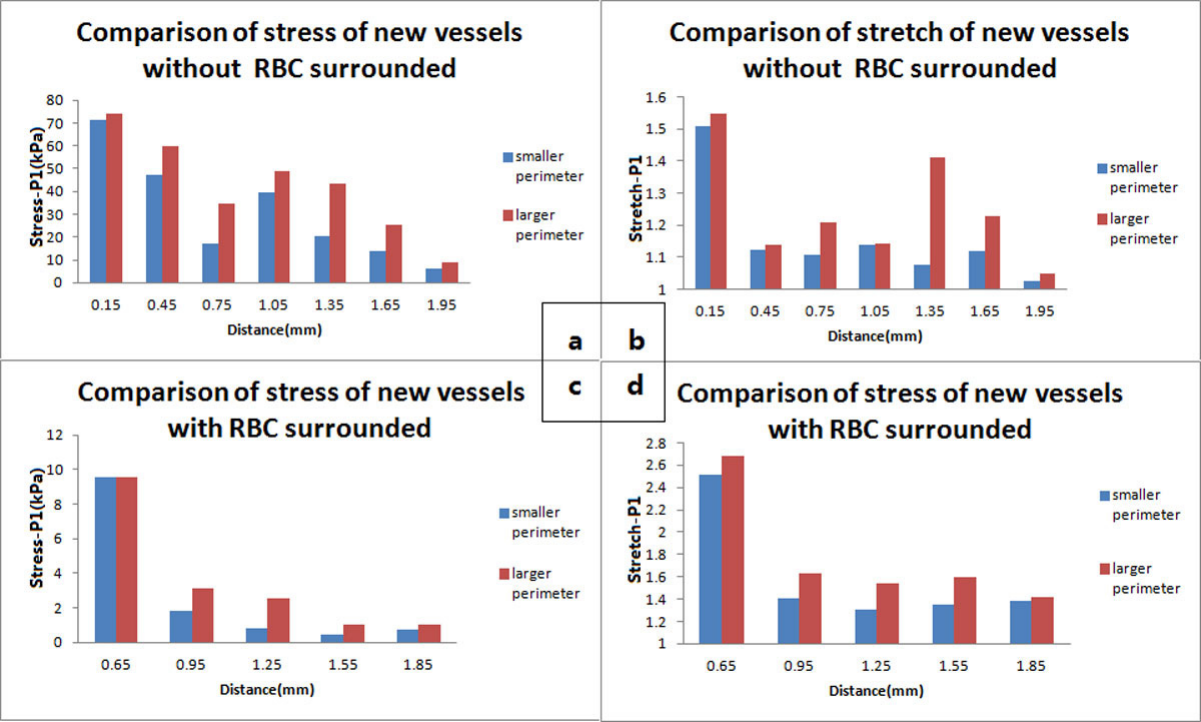
**Comparison of the stress and strain under different conditions**. (a) Comparison of stress (Stress-P1) of neovessels without red blood cells surrounded; (b) Comparison of stretch (Stretch-P1) of neovessels without red blood cells surrounded; (c) Comparison of stress (Stress-P1) of neovessels with red blood cells surrounded; (d) Comparison of stretch (Stretch-P1) of neovessels with red blood cells surrounded.

The sizes of neovessels in atherosclerotic plaques are different. According to the perimeter, neovessels are divided into two groups (one group with the larger perimeter than the average and the other group with the smaller perimeter than the average). The relationship between the mechanical condition and the perimeter for each neovessel is evaluated by comparing the Stress-P1 and Stretch-P1 of every two neovessels with different size and similar maximum curvature in the area with equal distance to the lumen. The number of neovessels ranges from 5 to 30 in each comparison group of the same distance to lumen. Figure 4 illustrates the result of the effect of the size on its mechanical condition.

As is shown in Figure 4, in the areas with the same distance to lumen, the larger the perimeter of neovessel is, the greater stress (Figure 4a) and deformation (Figure 4b) the neovessel suffers. The same result is observed in the area with-RBC surrounded and in area without-RBC surrounded (Figure 4c, d).

### Effect of the curvature

**Figure 5:**
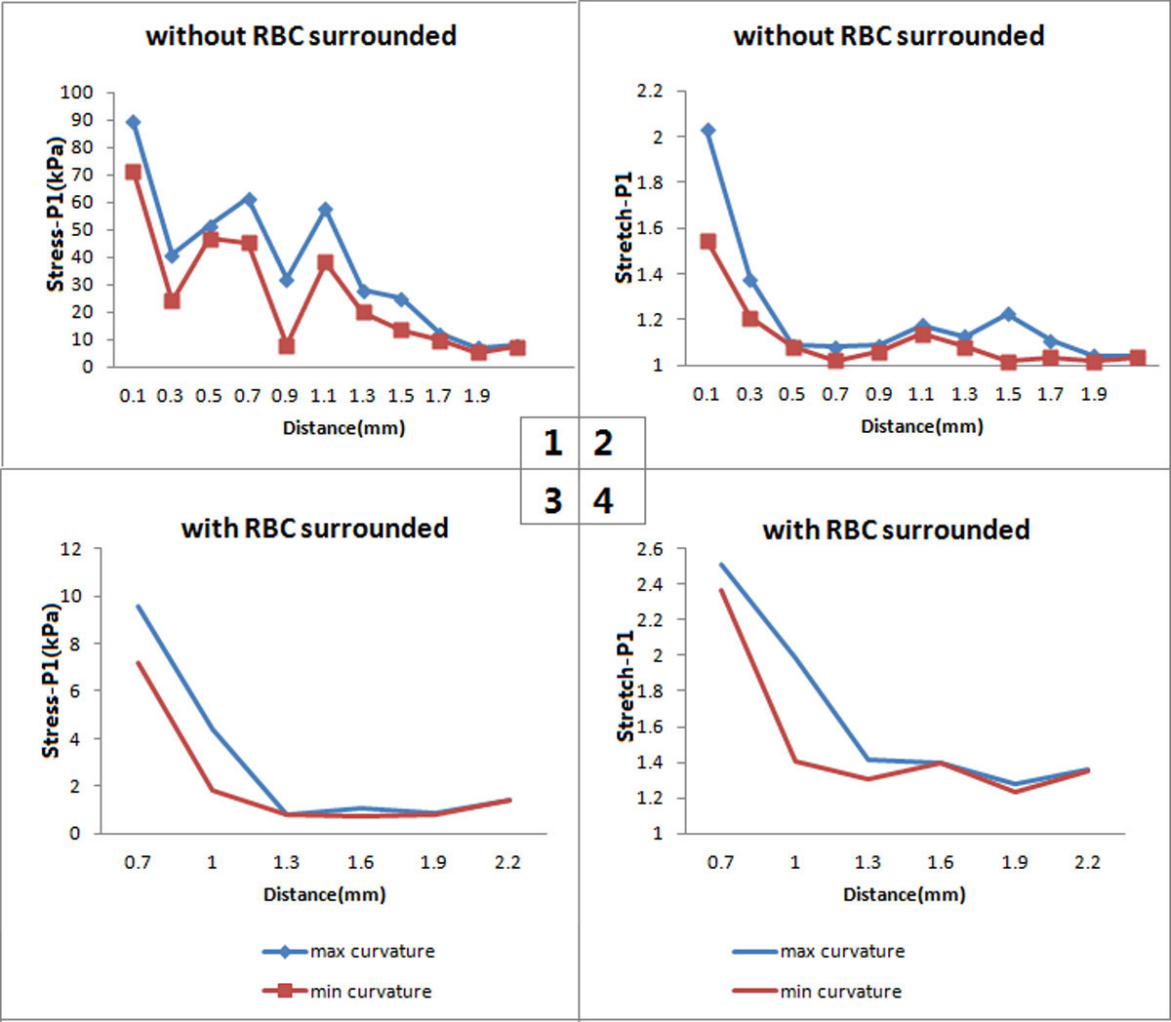
**The relationship between critical mechanical conditions around the neovessels and their curvature**. (a) Comparison of stress (Stress-P1) of neovessels without red blood cells surrounded; (b) Comparison of stretch (Stretch-P1) of neovessels without red blood cells surrounded; (c) Comparison of stress (Stress-P1) of neovessels with red blood cells surrounded; (d) Comparison of stretch (Stretch-P1) of neovessels with red blood cells surrounded.

Whether the mechanical situation of the neovessels will be changed if the curvature of the neovessel is different? By using the MATLAB to calculate the curvature of each neovessel, the neovessels with the same size and the same distance to the lumen were chosen to compare the mechanical conditions between the neovessels with the maximum and the minimum curvature. The results are shown in Figure 5.

Under the same conditions, the neovessel with larger curvature suffers greater stress and stretch in the area without-RBC (Figure 5a&5b). The same result can also be found in the area with-RBC (Figure 5c&5d).

### Effect of vessel material properties

In the abovementioned results, the neovessels are divided into two groups base on its location in IPH area or not (i.e., with-RBC and without-RBC). The neovessels in S1 (the neovessels in IPH area in S2 are too lack to get accurate analysis result) are chosen to compare the local maximum principal stress (Stress-P1) and stretch (Stretch-P1) between neovessels with and without RBC surrounded. Table 2 compares the local maximum stress and stretch between neovessels with and without-RBC surrounded.

**Table 2:** Comparison of the local maximum stress and stretch between neovessels with- and without-RBC surrounded.

Distance (mm)	0-0.6	0.6-1.2	1.2-1.8	1.8-
Stress-P1 (with-RBC) (kPa)	[12.92,89.29]	[5.46,61.33]	[6.23,43.40]	[1.55,25.26]
Stress-P1 (without-RBC) (kPa)	-	[0.63,10.08]	[0.44,3.14]	[0.42,1.87]
Stretch-P1 (with-RBC)	[1.08,2.03]	[1.02,1.23]	[1.02,1.41]	[1.01,1.14]
Stretch-P1 (without-RBC)	-	[1.22,2.68]	[1.26,1.79]	[1.28,1.49]

Under the pressure of carotid artery, the neovessels with-RBC surrounded undergo greater stretch than those without-RBC surrounded. The neovessels are more likely to deform when they locate in IPH area.

## Discussion

Histopathological examinations have revealed the association between IPH and the presence of neovessels [29-31]. Neovascularization is the process of generating neovessels mediated primarily by progenitor and/or endothelial cells leading to tube formation, resulting in a stabilized neovascular channel [29,32]. The neovessels are born of the outer membrane of nourishing and distributed from the epicardial fat to the plaque throughout vessel wall [33]. Blood components, such as RBC, neutrophils and other proinflammatory cells, may migrate from the bloodstream into the plaque because of the incomplete development of the vascular wall about angiogenesis [24,34,35]. Under this condition, the plaque is more likely to distort, and further develop IPH. In previous studies, investigators are more willing to discuss the impacts of the plaque size and stenotic degree, the inflammatory factors and the arterial pressure, and so on [3,28,36-38]. The structure of vulnerable plaque contains the following features: (a) large lipid core; (b) high density of macrophages; (c) low density of smooth muscle cells in the cap; (d) high tissue factor content; (e) thin plaque cap in which the collagen structure is disorganized [39]. However, the neovessels in the plaque should not be ignored [17,29,40].

The motivation of the present study is trying to explain the mechanisms about the neovessels in plaque from the biomechanical insights. As known to all, the rupture of plaque is mainly due to the stress and strain suffered, so two-dimensional models containing neovessels and other components were built according to the plaque slice. The main findings of finite element analysis are as follows.

(1) The stress and strain decrease with the increase of the distance between neovessel and lumen, which suggests that the carotid arterial pressure may play an important role in the deformation of neovessels. There is no direct association between the two groups of neovessels (i.e. one group in IPH area with-RBC surrounded and another group in area without-RBC surrounded), so we didn't discuss the comparison of stress and strain values of them. The neovessels in the plaque suffered a cyclic load with the pulsatile heartbeat. The stress and strain/stretch changes periodically. The neovessels may fatigue under cyclic loading, and those close to lumen are more dangerous.

(2)The neovessel with larger perimeter suffers much greater deformation, which indicates that the size is a significant factor for the fatigue of neovessels. The neovessels features high permeability and poor stability. Thus, the longer perimeter they have, the higher risk of rupture they face. Because the mechanical environments of the neovessels are related to integrated conditions including its shape, curvature, distance to the carotid lumen and the material properties of plaque, etc. We intended to show the result to express the influence of a single factor condition; however those figures in Figure 4 can not isolate the single factor while showing the total values of stress and stretch. We defined the neovessel with equal distance to lumen (per 0.3 mm) and similar curvature values (difference within ten times) for a group to investigate the effect of size on neovessels. We believe that the contradiction is due to the overall impact of the whole integrated factors.

(3) Under the same conditions, the neovessel with larger curvature suffers greater stress and stretch. The great curvature of neovessel with "sharp" edge generates stress concentration which is more easily to induce hemorrhage rupture.

(4) Those neovessels with-RBC surrounded undergo greater Stretch-P1 than those without-RBC surrounded. It indicates that the location of neovessels in plaque is also a significant factor for the deformation of neovessels. The hostile mechanical environment around neovessels may be concerned with the divulging of RBCs, which can be found around those neovessels suffered a large deformation. However, there is no significant difference of Stress-P1 between the groups with-RBC and without-RBC.

Although the angiogenesis can be identified as an effective means of increasing myocardial perfusion, the relationship between neovessels and plaque instability cannot be ignored yet. Our study demonstrates that neovessels in plaque rupture and hemorrhage may promote the plaque rupture, and increase the risk of stroke.

Some limitations in this study must be mentioned. First, only two samples with total 292 neovessels were modeled, which may not be enough for the strict verification to support our hypothesis. Some factors, such as the density of neovessels ought to be considered. Second, this study was a two-dimensional simulation, and the effect of the blood flow was not taken into account. The flow in the lumen and the week flow in the neovessels need to be considered for further hemodynamic calculation. In the process of staining, the lipid core occurred shedding in S2, so we ignored it when performing the numerical simulation analysis. But the results are within very small discrepancy and still support our forecast.

## Conclusions

Our hypothesis is that the mechanical situations of intraplaque neovessel are largely due to the complexity of biomechanical interactions. We suggest that there are large degrees of stress and deformation by the mechanical loading around the neovessels in plaque. By using the numerical method to analyze the mechanical conditions of neovessels in the plaque, we found that local mechanical conditions contribute to the neovessel damage and further IPH formation. Results show that those neovessels closer to the carotid lumen undergo larger stress and stretch. With the same distance to the carotid lumen, the longer is the perimeter of neovessels, the larger stress and the deformation are generated on the neovessels. Neovessels surrounded by red blood cells undergo a much larger stretch. Local mechanical conditions may result in the hemorrhage of neovessels and accelerate the rupture of plaque. The mechanical environments of the neovessels are related to its shape, curvature, distance to the carotid lumen and the material properties of plaque. The finding of this paper may be applied to evaluate the stability of plaque and provide a new way for the clinical assessment of stroke risk.

## Competing interests

Other than the grants listed in the acknowledgment section, the authors declare that they have no other competing interests.

## Ethics statement

All human data used in this study were obtained from consenting patients of the Neural Department of Internal Medicine in Beijing Tian Tan Hospital, and was approved by the hospital's Ethical Research Committee.

## Authors' contributions

All authors 1) have made substantial contributions to conception and design, or acquisition of data, or analysis and interpretation of data; 2) have been involved in drafting the manuscript or revising it critically for important intellectual content; and 3) have given final approval of the version to be published. Each author has participated sufficiently in the work to take public responsibility for appropriate portions of the content.

## Declarations

Publication of this article was paid with funding from National Natural Science Foundation of China (81171107).

This article has been published as part of *BioMedical Engineering OnLine *Volume 14 Supplement 1, 2015: Cardiovascular Disease and Vulnerable Plaque Biomechanics. The full contents of the supplement are available online at http://www.biomedical-engineering-online.com/supplements/14/S1
